# The Influence of External Forces on Wrist Proprioception

**DOI:** 10.3389/fnhum.2017.00440

**Published:** 2017-08-31

**Authors:** Francesca Marini, Sara Contu, Chris W. Antuvan, Pietro Morasso, Lorenzo Masia

**Affiliations:** ^1^Motor Learning and Robotic Rehabilitation Laboratory, Department of Robotics, Brain and Cognitive Sciences, Istituto Italiano di Tecnologia Genova, Italy; ^2^School of Mechanical and Aerospace Engineering, Nanyang Technological University Singapore, Singapore

**Keywords:** proprioception, wrist position sense, robotic rehabilitation, haptic interaction, external forces

## Abstract

Proprioception combines information from cutaneous, joint, tendon, and muscle receptors for maintaining a reliable internal body image. However, it is still a matter of debate, in both neurophysiology and psychology, to what extent such body image is modified or distorted by a changing haptic environment. In particular, what is worth investigating is the contribution of external forces on our perception of body and joint configuration. The proprioceptive acuity of fifteen young participants was tested with a Joint Position Matching (JPM) task, performed with the dominant wrist under five different external forces, in order to understand to what extent they affect proprioceptive acuity. Results show that accuracy and precision in target matching do not change in a significant manner as a function of the loading condition, suggesting that the multi-sensory integration process is indeed capable of discriminating different sub-modalities of proprioception, namely the joint position sense and the sense of force. Furthermore, results indicate a preference for target undershooting when movements are performed in a viscous or high resistive force field, rather than passive or null fields in which subjects did not show any predominance for under/over estimation of their position.

## 1. Introduction

Proprioception is the sensory stream responsible for the conscious perception of body position (joint position sense, JPS) and movement (kinaesthesia), for the sense of tension or force, the sense of effort, and the sense of balance. Deformation of skin, muscles, tendons, fascia, and joint capsule during motion, are encoded by mechanically sensitive receptors that innervate body tissues. Afferents from these receptors project to the cortex for conscious perception of action (Proske and Gandevia, 2012).

Allowing humans to control their limbs without directly looking at them, proprioception is necessary for the accomplishment of most of the activities of daily living, and it provides crucial information for successful task completion (Touzalin-Chretien et al., 2010) to maintain an updated body image or body schema (Morasso et al., 2015).

In our daily life, this schema is perturbed by different external dynamics that require us to produce various motor commands: we are able, for instance, to grasp and put a filled or an empty cup on the table with the same accuracy.

There is so far, an extensive literature on the effect of loads on human movements and several motor control studies provide evidence of how we only respond to environmental forces if they affect task success, according to the “minimum intervention principle” (Todorov and Jordan, 2002) and the “uncontrolled manifold hypothesis” (Scholz and Schöner, 1999; Latash et al., 2007; Latash, 2013). In particular, in case of reaching tasks with loose spatial and temporal constraints, humans change their kinematics before being pushed by a force that had no bearing on the task completion (Cashaback et al., 2015). Furthermore, the human motor system appears to be able to adapt its representation of dynamics during learning of a motor task (Shadmehr and Mussa-Ivaldi, 1994). There is indeed some evidence for independent learning of internal models for kinematic and dynamic control of reaching (Ghez et al., 1999) showing the ability of the central nervous system to adapt to unstable dynamics (Burdet et al., 2001). It is only in recent years that research is investigating correlation between motor control and external dynamics to understand the influence of external forces on human position sense (Kuling et al., 2013). Yet, up to date, while somatosensory plasticity and changes in proprioception after force field motor adaptation have been intensely investigated (Ostry et al., 2010), information about the relationship between force and related proprioceptive accuracy is still limited. Only few contrasting results have been obtained when measuring different joints repositioning errors in the presence or after the interaction with external forces. Recent findings show that external forces inducing contraction followed by relaxation of a muscle, without changing its length affects the spindle discharge rates and modifies the perceived limb position (Gregory et al., 1988; Allen et al., 2007). Additional evidence in support to a detrimental effect of external forces on proprioception comes from the observation that subjects make higher errors both in active and passive position matching after exercise in presence of external forces of one arm (Saxton et al., 1995; Voight et al., 1996; Brockett et al., 1997; Lee et al., 2003; Walsh et al., 2004). Conversely, other studies propose that when a muscle becomes active, spindle signals, which arise as a result of fusimotor activity, are subtracted out centrally (McCloskey and Torda, 1975; McCloskey et al., 1983) by means of an efference copy of the motor command (Holst and Mittelstaedt, 1950) and proprioception does not appear to be affected by external dynamics. To the best of our knowledge, no studies testing the effect of different force conditions on JPS of the wrist have been conducted, and literature about correlation between proprioception and external forces is, in general, still very limited (Kuling et al., 2013).

In order to fill this gap, in the present study, we measured proprioceptive acuity of the wrist with a position matching task performed in five different loading conditions.

Overall, the results indicate that accuracy and precision of proprioception are robust under external forces, but highlight a preference for target undershooting when movements are performed in viscous or high resistive force field, rather than passive o null fields in which subjects did not show any predominance for under/over estimation of their position. These results suggest that the influence of force on proprioception may be coded centrally, but peripheral receptors have a strong influence on the final perception as well.

## 2. Methods

### 2.1. Participants

Fifteen right-handed subjects, with no history of neuromuscular disorders and naive to the task, participated in the study. All the participants were young males (mean age 27.73 ± 2.91 *SD* years) with similar biomechanics characteristics. Handedness of all participants was assessed using the Edinburgh Handedness Inventory (Oldfield, 1971), and the study was approved by the Institutional Review Board at Nanyang Technological University and was performed at the School of Mechanical and Aerospace Engineering of the same university. Written informed consent was obtained from the participants of this study.

### 2.2. Apparatus

**Figure 1:**
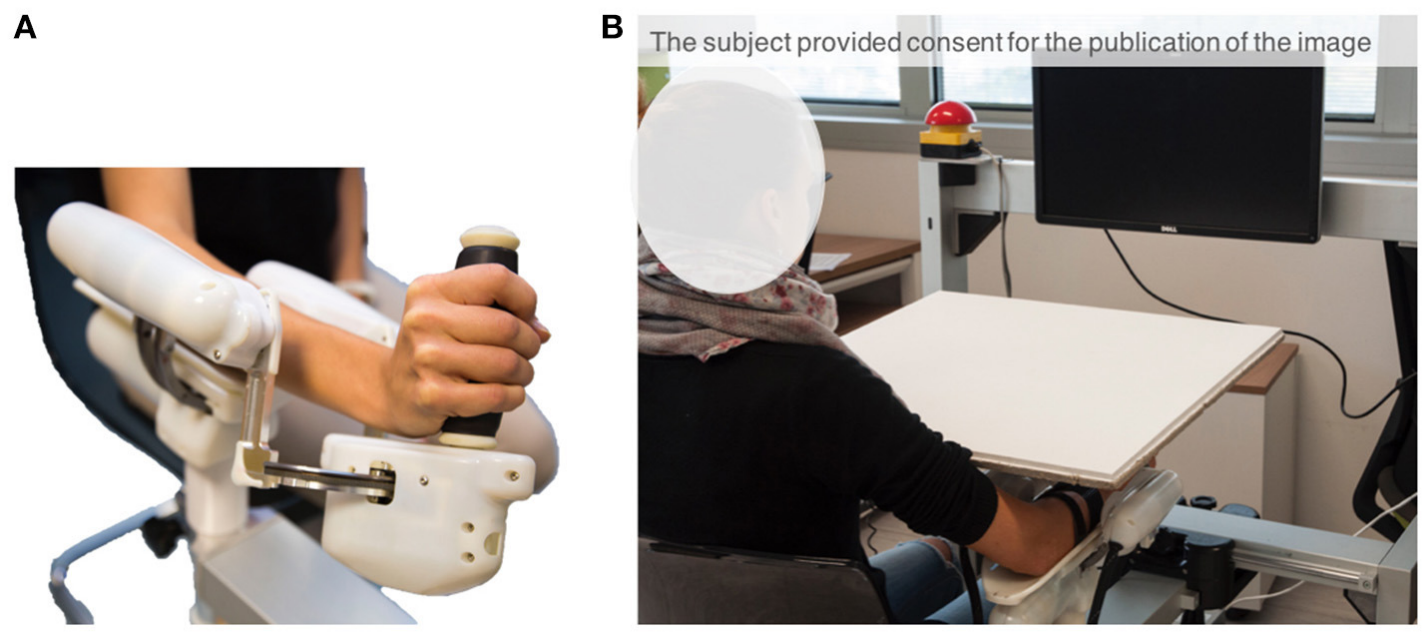
**(A)** Wrist robotic device. **(B)** Experimental set-up comprising the robot and the computer screen. Subject's wrist and the robot were visually occluded through a board, so as to engage only the sense of proprioception when performing the task.

The experimental apparatus (Figure 1A) consisted of a three-degrees of freedom (DoF) wrist manipulandum designed for motor control studies and rehabilitation (Masia et al., 2009; Squeri et al., 2014). The roboti allowed movements along the three wrist DoFs: flexion/extension (FE: ±70°), radial/ulnar deviation (RUD: ±35°), and pronation/supination (PS: ±80°), for almost the full Range of Motion (RoM) of the human wrist. The device is provided with four Maxon R brushless motors (one EC-45 flat 70 W direct drive for FE, two ECi-40 70 W with 14:1 gearhead for AA, and one ECi-40 70 W with 3.7:1 gearhead for PS) that support an accurate haptic rendering and compensation of weight and inertia of the device. The maximum continuous torque levels provided by the motors are: 1.53 Nm for FE, 1.63 Nm for RUD, and 2.77 Nm for PS. Four high resolution incremental encoders are embedded in the respective motor to measure angular rotations of the three DoFs. The mechanical transparency of the device was enhanced by a control algorithm, for inertia and gravity compensation, intended to reduce force and effort during the task and to avoid involvement of other muscles except those except those naturally involved by the task. Furthermore, the handle of the device was carefully designed in order to allow anatomical grasp and minimize stretching of the fingers that may lead to exaggerated activation of flexor/extensor muscles during the active phase of the matching task. Data Collection Frequency was set at 100 Hz.

The apparatus includes a screen, placed approximately 50 cm away from the subject, in order to provide visual feedback relevant for the tasks: target location is represented on the screen as a red cursor and the wrist rotation as a moving white cursor (Figure 1B). Only flexion/extension movements were considered in this study. The other two DoFs were constrained to their neutral position by active control.

### 2.3. Task and procedure

Participants sat beside the robotic device, facing forward, and placed their forearm on the rigid cast of the robot, with the elbow flexed to about 120° (Figure 1B). After ensuring the correct alignment between the axes of the robotic system and the wrist's anatomical ones, the subjects' forearm was firmly strapped to the mechanical support. This ensured repeatability of wrist positioning and limited inter-trial variability. It also avoided joints misalignment and unwanted relative movements during the entire duration of the task. The subject grasped the handle of the robot which only allowed movements along the flexion/extension direction. The vision of the wrist and the robot was visually occluded by a board, so as to limit sensory feedback during the task only to proprioceptive signals (Figure 1B).

**Figure 2:**
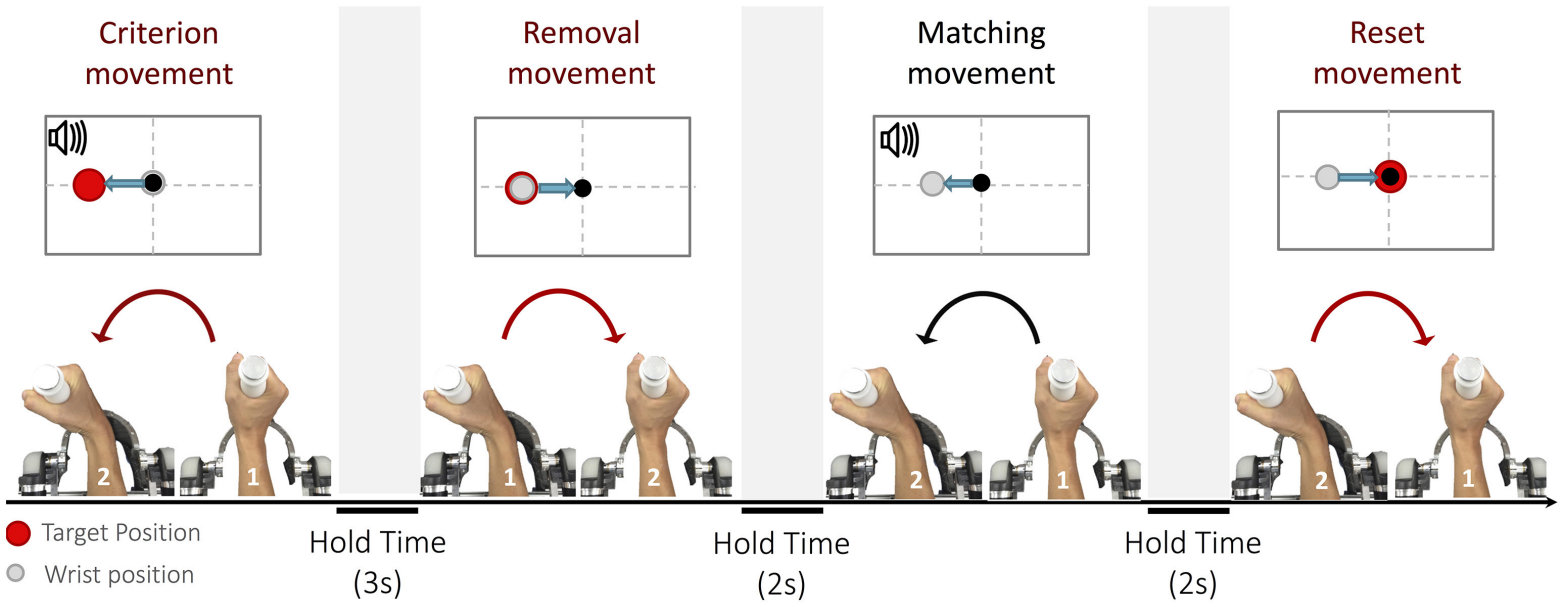
The Joint Position Matching task: Each trial is broken down into four parts: (1) criterion movement toward the proprioceptive target, (2) removal movement from the proprioceptive target, (3) matching movement, (4) and reset movement to the neutral position. Depending on the loading condition, the four movements can be performed either actively or passively.

The purpose of the experiments was to ascertain to which extent the position sense is influenced by loading conditions that require different activation patterns of muscles to reach the same position (Semprini et al., 2016). In particular, proprioceptive acuity was evaluated with an ipsilateral joint position matching task (JPM) (Goble, 2010). In this test, each trial consisted of four phases, characterized by specific movements (Marini et al., 2017): (1) a *criterion movement*, (2) a *removal movement*, (3) a *matching movement* and a final *reset movement* back to the starting position (see Figure 2). During the *criterion movement*, the subject wrist reached, through an active or passive action, a pre-determined position, thus allowing him to memorize, via proprioceptive feedback, the target position (shortly, proprioceptive target); the purpose of the *matching movement* was to actively replicate the target position.

Participants were instructed to concentrate only on the end location of the *criterion movement* and replicate it as accurate as possible during the *matching movement*.

To investigate whether proprioceptive acuity is biased by external forces, proprioceptive targets were presented in five different dynamic conditions: Passive (P), High Resistive (HR), Low Resistive (LR), No Force (NF), and Viscous (V).

While the elastic field exerts a counter force toward a certain position linearly proportional to the distance from that position, the viscous field resist movement linearly with respect to velocity. Accordingly, the force fields applied in the HR and LR conditions exerted a counter force persistently active throughout a movement, and the force field applied during the V condition was transitorily active during the matching movements and tended to vanish near the target.

#### 2.3.1. Passive condition (P)

In this condition the *criterion movement* was a passive displacement by the robot to the proprioceptive target, followed by a steady maintenance the position for a period of 3 s, during which subjects were instructed to memorize the position. The passive motion was provided by an elastic control torque that attracted the wrist to the target:

(1) $$\begin{array}{r} T_{P}=K_{P}\left(\theta_{T}-\theta_{W}\right) \end{array}$$

where θ_*W*_ is wrist's position, θ_*T*_ is the proprioceptive target position, and *K*_*P*_ = 5*N*/*deg* is the stiffness of the elastic field. In this condition, the *criterion movement* did not require any active movement from the subject as the robot applied the needed torque to displace the wrist until it reached the target position, and no visual feedback of target or wrist position was provided. After the 3 s dwell time, the subject's wrist was passively moved back by the robot to the neutral configuration (*removal movement*) and an auditory cue suggested to the subjects they could start the *matching movement*. In this phase subjects were requested to reproduce the previously memorized proprioceptive target, as accurately as possible, without any robot assistance and/or visual feedback. Finally, the trial was terminated by a passive displacement to the neutral position.

#### 2.3.2. Resistive conditions (HR and LR)

In this condition a constant torque was persistently active that pushed the hand away from the target position (Equation 2).

(2) $$\begin{array}{r} \{\begin{array}{c} T_{R}=-T_{HR}(\theta_{T}-\theta_{0})/|\theta_{T}-\theta_{0}|\\ T_{R}=-T_{LR}(\theta_{T}-\theta_{0})/|\theta_{T}-\theta_{0}| \end{array} \end{array}$$

where: θ_*T*_ is the proprioceptive target position, θ_0_ is the neutral wrist position, *T*_*HR*_ = 0.450*Nm* is the intensity of the “High Resistance” field and *T*_*LR*_ = 0.225*Nm* is the intensity of the “Low Resistance” field (consider that the maximum torque delivered by the motors for the FE movements is 1.53 Nm). In this condition, during the *criterion movement*, target and wrist positions were both displayed on the screen and thus the subject had to acquire the proprioceptive target information while pushing against the resistive force. As in the P condition, the criterion phase was terminated after the subject succeeded to maintain the target position for 3 s. After a passive *removal movement*, the following *matching movement* was performed actively, against the same resistance, but without any visual feedback.

#### 2.3.3. Viscous condition (V)

In this condition a torque was persistently applied that opposed active movements and was proportional to the wrist speed:

(3) $$\begin{array}{r} T_{V}=-B\overset{\cdot }{\theta_{W}} \end{array}$$

where $\dot{\theta_{W}}$ is the wrist speed and *B* = 0.01 *sNm*/*deg* is the coefficient of viscosity. In this condition, during the *criterion movement* subjects were requested to actively reach, with visual feedback, the target position. After the *removal movement*, the following *matching movement* had to be performed against the same viscous field without any visual feedback.

#### 2.3.4. No-force condition (NF)

In this condition the robotic device did not provide any torque neither during the *criterion* nor during the *matching movement*. During the *criterion movement*, the subjects were instructed to actively achieve the target position under visual guidance. After the dwell time and the following *removal movement*, the *matching movement* could start, without any visual or haptic feedback.

**Figure 3:**
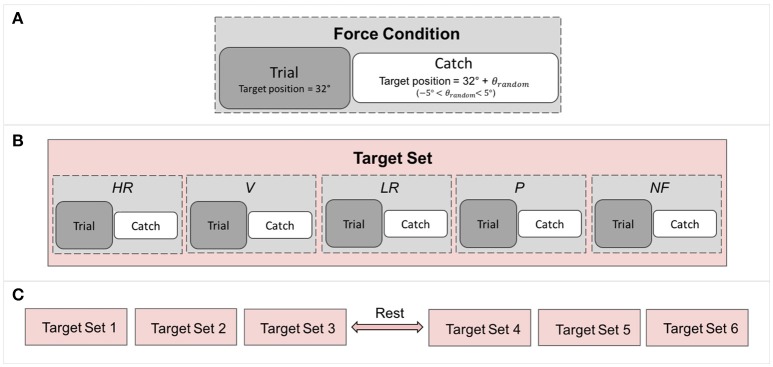
Breakdown of the experiment: **(A)** Each force condition comprises a trial followed by a catch trial in which the target position is randomized to control for subject adaptation. **(B)** A target set contains all the force condition. **(C)** The experiment comprises 6 target sets, resulting in 6 repetitions of each force conditions.

The type of feedback (Visual and/or haptic) for each condition is summarized in Table 1; the breakdown of each experimental trial is shown in Figure 3. In all the five conditions, the matching movement was considered completed when wrist's speed was lower than a 2°/s threshold for more than 2 s.

**Table 1:** Feedback (haptic and visual) during *reaching* and *matching* movement in the five force conditions.

	**Criterion movement**		**Matching movement**		**Removal/Reset movement**	
	**Haptic feedback**	**Visual feedback**	**Haptic feedback**	**Visual feedback**	**Haptic feedback**	**Visual feedback**
HR Resistive elastic force	✓ Constant opposing torque	✓	✓ Constant opposing torque		✓ Constant opposing torque	✓
LR Resistive elastic force	✓ Constant opposing torque	✓	✓ Constant opposing torque		✓ Constant opposing torque	✓
V Resistive viscous force	✓ Speed-dependent opposing torque	✓	✓ Speed-dependent opposing torque		✓ Speed-dependent opposing torque	✓
P Passive elastic force	✓ Elastic assistive torque				✓ Elastic assistive torque	
NF no force		✓				✓

Proprioceptive targets were located at 32° of flexion and trials were pseudo randomized across conditions (Figure 3) and presented to each subject with the same order. To avoid subjects being adapted to the task and target location, catch trials were introduced in which targets were located as follow:

(4) $$\begin{array}{r} TG=32\pm TG_{shift} \end{array}$$

where *TG*_*shift*_ randomly varied from 1° to 2.5°. A catch trial followed each trial, as depicted in Figure 3. Each trial was repeated six times for conditions for a total of 60 matchings (30 trials + 30 catch trials) lasting about 45 min. A pause of 10 min in between of the experiment let subjects rest and refocus their attention on the task.

### 2.4. Performance measures

Wrist joint rotations, recorded from the robot's incremental encoders, were post-processed by a third-order Savitzky-Golay low-pass filter (cut-off frequency of 10 Hz) and converted into angular displacements from the direct kinematics of the robot. In order to characterize different aspects of proprioceptive acuity (Schmidt and Lee, 1988; Dukelow et al., 2010; Marini et al., 2016a) three different indicators of performance were computed in the five loading conditions: the *Matching Error* (*ME*), the *Matching Variability (MV)* and the *Matching Bias* (*MB*); moreover, the *Loading Torque* (*LT*) was measured to correlate the performance indicators to the force magnitude applied to the wrist during matching over the different conditions.

#### 2.4.1. *Matching error (ME)*:

For each loading condition, it is computed as the average absolute discrepancy between the target position and the average wrist position during the dwell time at the end of the matching movements:

(5) $$\begin{array}{r} \text{ME}=\frac{\sum_{i=1:N}|\theta_{i}-\theta_{T}|}{N} \end{array}$$

where θ_*i*_ is the wrist final position at the end of the matching movement of the *i*-trial (average position in the 3 s holding time), θ_*T*_ is the proprioceptive target position, and *N* = 6 is the number of trials.

#### 2.4.2. *Matching Variability (MV)*:

It is computed as the standard deviation across the *N* = 6 trials of the wrist position (θ_*i*_) at the end of the matching movement:

(6) $$\begin{array}{r} \text{MV}=StD\left(\theta_{i}-\theta_{T}\right) \end{array}$$

#### 2.4.3. *Matching bias (MB)*:

It is similar to *ME* with the difference that it takes into account the sign of the discrepancy between the target position and the average wrist position:

(7) $$\begin{array}{r} \text{MB}=\frac{\sum_{i=1:N}\left(\theta_{i}-\theta_{T}\right)}{N} \end{array}$$

#### 2.4.4. *The loading torque (LT)*:

It is the peak value of the torque applied to the wrist during the matching movement averaged across the *N* = 6 trials.

As already noted, the performance indicators are supposed to characterize different aspects of proprioceptive acuity: *MV* measures the degree of repeatability of the matching actions performed by the subjects, independent of their accuracy, i.e., the distance between the target and the matched position; moreover, *MB* expresses the systematic tendency to over- or under-estimate the target position.

### 2.5. Statistical analysis

Due to the small sample size the Shapiro–Wilks test was used to ensure that all variables fit the Gaussian distribution and revealed that data did not meet the assumption of normality. Therefore, non-parametric analyses were chosen accordingly. Specifically, to examine differences among the force conditions, a Friedman's test for repeated measures was performed on group data for *ME* and *MB* (level of significance was set at *p* = 0.05). In case of significance, *post-hoc* analysis with Wilcoxon signed-rank test was conducted for pairwise comparison, with a Bonferroni correction for multiple comparisons, resulting in a significance level set at *p* < 0.005. For *MV* (standard deviation across the *N* = 6 trials of wrist final position), a non-parametric Kruskal–Wallis test was conducted to investigate the effect of the force condition.

Finally, a single sample *t*-test was conducted between zero and the *EB* to investigate significance of target under/overshooting.

Strength and direction of the relationship between the *Loading torque* (*LT*) and the *Matching bias* (*MB*) were investigated by measuring their correlation coefficient, and the corresponding least-squares line was found.

## 3. Results

**Figure 4:**
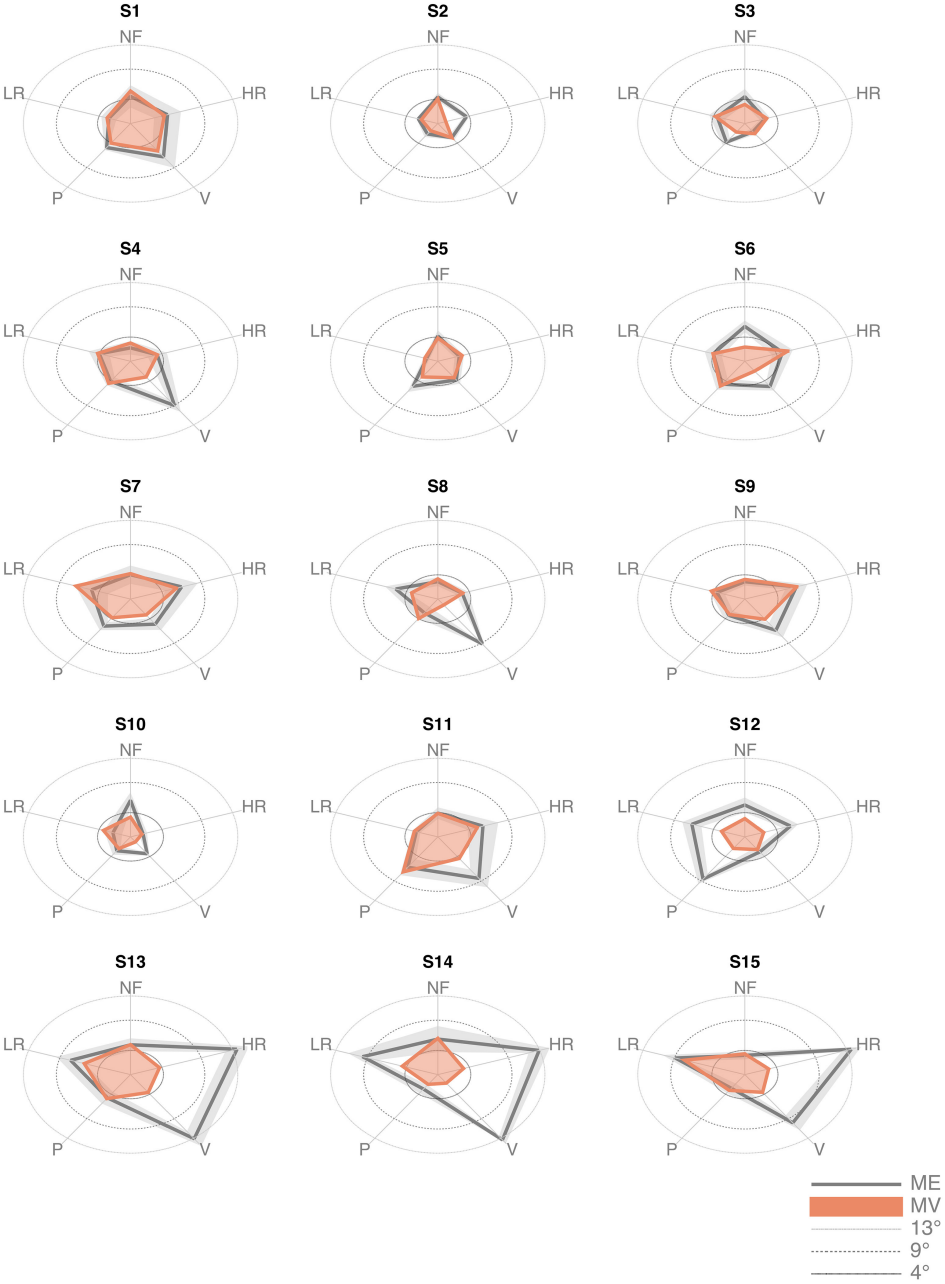
*Matching error* (Mean and standard deviation across the 6 trials) and *Matching Variability* (standard deviation of the matched position across the 6 trials) of the 15 subjects, in the five force conditions.

The inspection of the data revealed that three subjects (S13, S14, and S15) exhibited much larger values of the *ME* indicator in the high-load conditions (HR and V), as shown in Figure 4. Data from these subjects were excluded from the analysis. Indeed, we questioned them specifically and we discovered that they did not really understand the task, in the sense that they did not focus primarily on the detection and memorization of the target position, as requested, but they aimed at overcoming the resisting torques by maximizing the level of their muscle co-contraction.

Results from the analysis of the remaining 12 subjects are shown in Table 2, which reports mean and standard error of the 4 indicators (*ME, MV, MB*, and *LT)* in the five loading conditions (HR, NF, LR, P, V), respectively.

**Table 2:** *Matching error* and *Error variability* (mean and standard error of the 12 subjects) in High Resistive (HR), No-Force (NF), Low Resistive (LR), Passive (P), and Viscous (V) condition.

	**HR**	**NF**	**LR**	**P**	**V**
Matching error (ME)	4.26 ± 0.46°	4.21 ± 0.31°	3.65 ± 0.41°	4.51 ± 0.49°	5.62 ± 0.21°
Matching variability (MV)	4.04 ± 0.53°	3.81 ± 0.20°	3.84 ± 0.41°	3.87 ± 0.48°	3.39 ± 0.45°
Matching bias (MB)	−1.61 ± 0.95°	1.96 ± 0.90°	0.15 ± 0.85°	1.22 ± 1.01°	−4.93 ± 0.97°
Loading torque (LT)	0.450*Nm*	0*Nm*	0.225*Nm*	0*Nm*	0.52 ± 0.04*Nm*

**Figure 5:**
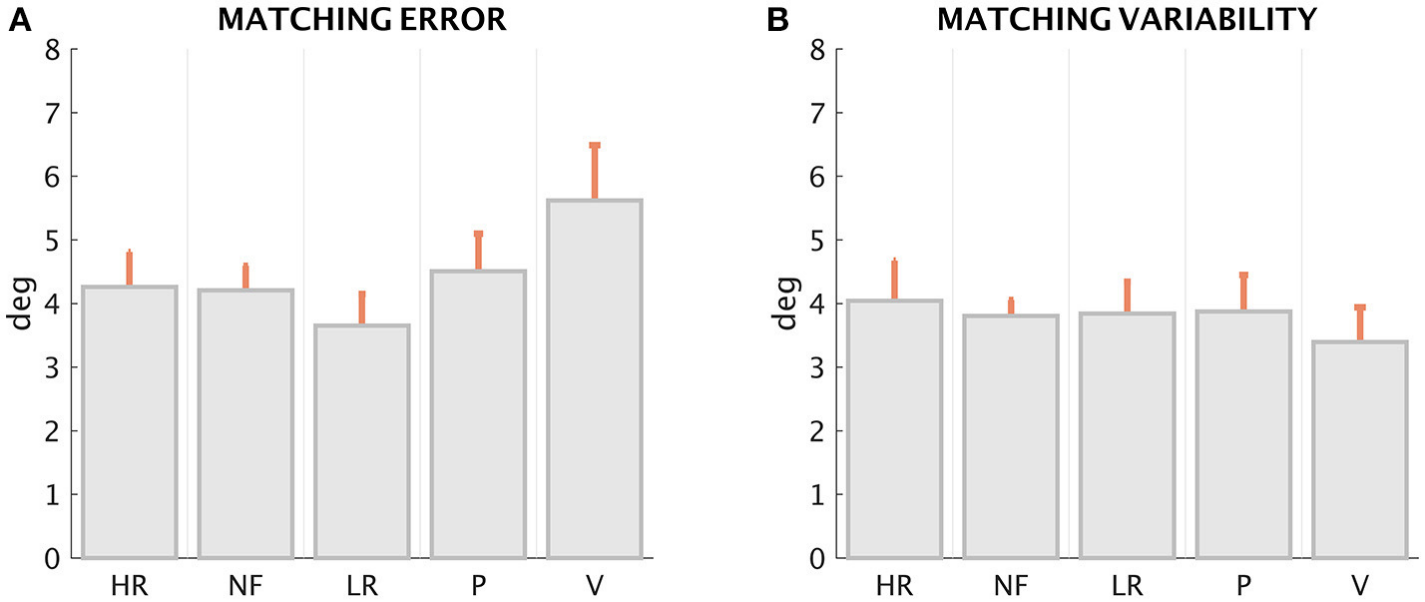
*Matching error* **(A)** and *Matching variability* **(B)**, mean ± SE of the 12 subjects, in the five loading conditions.

The first result (Figures 5A,B for *ME* and *MV*, respectively), suggested by the inspection of the patterns displayed in Figure 4, is that *ME* and *MV* do not appear to be affected in a significant manner by the loading conditions. This is confirmed by the statistical analysis performed with the Friedman's test for *ME*, which revealed a significant effect of force condition [χ^2^(*N* = 72, *df* = 4) = 13.62, *p* = 0.0086]. The *Post-hoc* analysis revealed a significant difference of the *ME* in the LR and V condition (*z* = 3.59, *p* = 0.0003). The non-parametric Kruskal–Wallis test revealed no significant differences among conditions for *MV* (χ^2^ = 0.67, *df* = 4, *p* = 0.9554; Figure 5).

The analysis of the *MB* hints at a slightly different picture. The Friedman's test reported a significant effect of force condition on the EB values over repetitions [χ^2^(*N* = 72, *df* = 4) = 75.19, *p* =< 0.001]. The with Wilcoxon signed-rank *post-hoc* test revealed significant differences between the viscous condition (V) and the other five conditions: (*MB*(*V*) − *MB*(*HR*) : *z* = 3.74, *p* = 0.0002; *MB*(*V*) − *MB*(*NF*) : *z* = 6.41, *p* < 0.0001; *MB*(*V*) − *MB*(*LR*) : *z* = 5.73, *p* = < 0.0001; *MB*(*V*) − *MB*(*P*) : *z* = 6.01, *p* < 0.0001). Similarly, the difference of the MB between the HR and in the NF conditions and between the MB in the HR and in the P conditions resulted significant: (*MB*(*HR*) − *MB*(*NF*) : *z* = 5.07, *p* < 0.0001; *MB*(*HR*) − *MB*(*P*) : *z* = 3.88, *p* = 0,0001).

**Figure 6:**
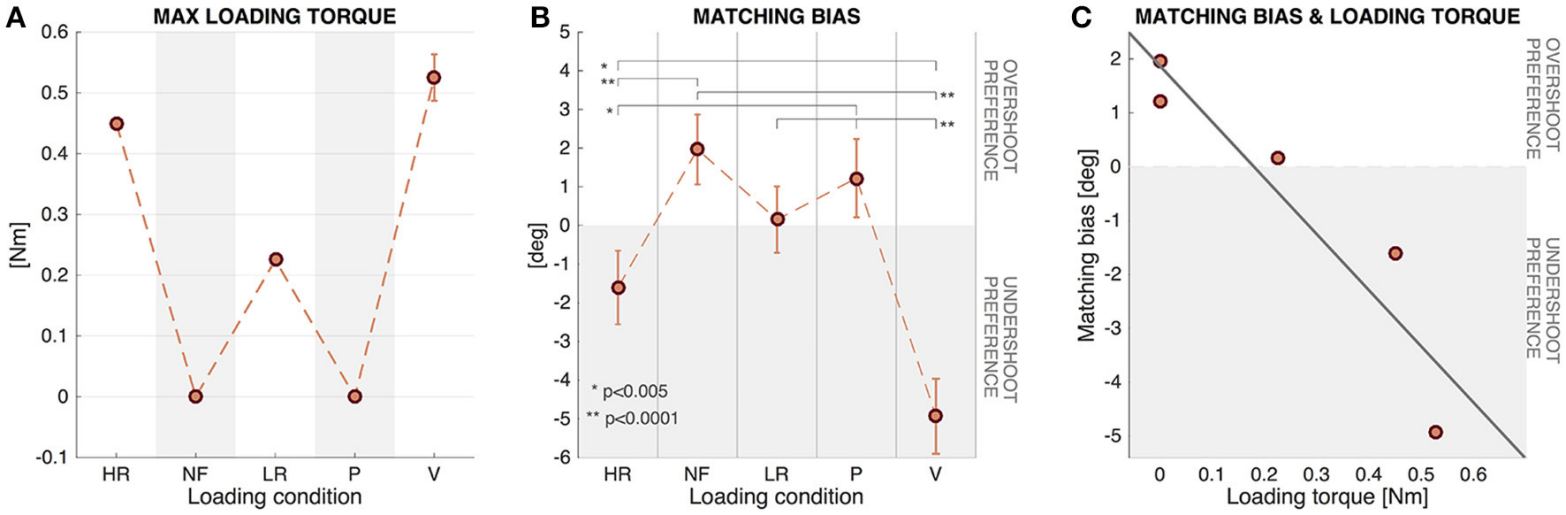
**(A)** Maximum value of loading torques (LT) experienced during the matching movements. Mean and SE of the 12 participants. **(B)** Matching Bias in the five different force conditions (Mean and SE of the 12 participants). Positive values indicate tendency for target overshooting and negative values tendency to undershoot. **(C)** Relationship between Matching Bias and Loading torque for the 12 subjects. Regression line corresponding to the scatter plot of the two variables.

Figure 6B shows that in the HR and V conditions, characterized by a high loading level, there was a tendency to undershoot the presented proprioceptive targets: this tendency is indeed significant according to the single sample *t*-test for both HR [*t*_(71)_ = -2.59, *p* = 0.0116] and V [*t*_(71)_ = -8.66, *p* < 0, 001] conditions. Conversely, in the NF condition, where EB was higher than zero (see Table 2 and Figure 6B), the single sample *t*-test revealed a significant difference from zero highlighting a significant tendency for target overshooting [*t*_(71)_ = 3.51, *p* = 0.0008]. The difference between the EB in the LR and P conditions with respect to zero was found to be not significant, suggesting that errors in these cases were approximately bias-free.

Figure 6A shows the LT experienced during the *matching movements* in the different conditions: No load was applied to the wrist in the NF and P conditions (*LT* = 0*Nm*); in the HR condition a constant value of resistive torque was applied (*LT* = 0.450*Nm*); in the LR condition the constant value was *LT* = 0.225*Nm*; in the V condition the load was variable, with a final value *LT*_*final*_ = 0*Nm* and a peak *LT*_*peak*_ = 0.52 ± 0.04*Nm*, dependent on the speed profile of the *matching movement*.

Linear regression analysis was used to develop a model for predicting subjects' behavior in terms of *Matching Bias* (MB) based on the external *Loading torque* (LT) experienced. These two variables resulted to be highly correlated (correlation coefficient = −0.93), and the least squares fit line (Equation 8 and shown in Figure 6C) was found to be significant [*F*_(1, 3)_ = 19.32, *p* = 0.0218], with an *R*^2^ = 0.86 highlighting a strong downhill linear relationship.

(8) $$\begin{array}{r} MB\left(LT\right)=-10.20LT+1.85 \end{array}$$

## 4. Discussion

In the present work, we investigated the influence of five different types of force fields on wrist joint proprioception in 15 healthy subjects. Results showed that accuracy and precision did not change as a function of loading. However, resistive and viscous force fields resulted in a tendency to slightly undershooting the target.

Proprioception is the sense of the relative position of neighboring parts of the body and strength of effort being employed in movement.

Position sense and sense of effort area critical components for the correct execution of activities of daily living. Several conditions can result in proprioceptive impairments leading to a reduced functional independence. Stroke in the post-central gyrus, supramarginal, angular, pre-central, and superior temporal gyri and insula can affect kinesthetic processing (Kenzie et al., 2016) and lesions in the thalamus, internal capsule, and post-central gyrus have been associated with “abnormal” position sense (Tong et al., 2010). The sense of effort can result defective in subjects affected by dystonia (Carment et al., 2017).

The different sensory sub-modalities, in particular, joint position sense (JPS) and sense of force should be at least grossly independent, in order to be functionally effective in the course of skilled control of action. However, they originate from the same multi-sensory integration process and it is somehow surprising that the CNS is indeed able to single out the different components.

The joint position sense is typically evaluated by mean of JPM tasks, while the sense of tension or force is commonly assessed using a Force Reproduction protocol (FR) (Jones and Hunter, 1983, 1990; Brockett et al., 1997).

While JPM tasks are usually performed in the absence of external forces (Goble, 2010; Marini et al., 2016b) and FR protocols involve an isometric test (Jones and Hunter, 1983), in which the force exertion is not accompanied by a joint rotation, many activities of daily living are performed in presence of variations of both position and force. As a matter of fact, JPM and FR protocols were found to be equally reliable measures of proprioceptive sub-modalities in the shoulder (Dover and Powers, 2003) but how these sub-modalities interact is still unclear. As a consequence, if and how the brain is capable of singling out the position-dependent and force-dependent aspects of proprioception was the object of this study and, in particular, we evaluated the robustness of JPS under the influence of force in different loading conditions.

It is quite evident that a strong influence would grossly impair the human capacity to carry out skilled tasks in a variety of environmental conditions without having to rely too much on the visual feedback.

The main result is consistent with the requirement of functional robustness of JPS formulated above. We found indeed a general consistent acuity in five different force fields with a constant trend of *matching accuracy* and *variability* across the subject population that did not differ in presence of the different force fields. This could be due to the fact that different types of ascending and descending signals could have contributed to joint position sense in the presence of external forces. While muscle receptors are known to be a prominent determinant of joint position sense, information from these peripheral receptors is integrated with signals of central origin (motor command) to provide information about force sense. When a muscle becomes active, spindle signals, which arise as a result of fusimotor activity, are subtracted out centrally (McCloskey et al., 1983; McCloskey, 2011) by means of an efference copy of the motor command (Holst and Mittelstaedt, 1950). The central command, or sense of effort, could, therefore, have provided primary information for the recognition of the target position in the presence of external forces responsible for decreasing the sensitivity of peripheral receptors.

Moreover, it is assumed that there is a link between motor and sensory areas in the cerebral cortex (Brooks et al., 2013), and the degree of activation of motor areas is directly expressed by the effort sensation. As the muscle is weakened by fatigue or paralysis, or has to deal with a resistive force field, the neuromuscular commands must be increased in order to achieve the desired target position; as a consequence, the effort sensation is increased (Jones and Hunter, 1990; McCloskey, 2011).

Yet, although it is clearly positive that similar trends were consistently detected in the whole population of subjects for the various force conditions, it is also worth mentioning that we detected subjects specific trends for both *matching error* and *matching variability*. This result supports the importance of peripheral signals to the sense of muscle force (Luu et al., 2011), interpreting peripheral contribution, not as a pure exafference, but rather as a reafference, generated in response to the motor command. The different subjects' behavior could be also related to the importance of intrinsic muscle properties. For instance, each muscle has its own length-tension properties and different force generator capabilities which vary among subjects, such as receptor density and muscle spindles presence.

We also found a higher standard deviation in the *matching error* especially in the case of high-resistive and viscous conditions. We believe that this difference can be attributed to the fact that forces levels were equal for all the subjects and were not tuned according to their maximum voluntary contraction. The same outcome characterizes also the *Matching bias*: we found a tendency to undershoot target positions only during the *high resistive* and the *viscous* force fields and, vice versa, a tendency to overshoot in the *no force* and *passive* conditions while in case of *low resistive* force field the error resulted to be bias free. Furthermore, the model resulting from the linear fitting resulted to be highly descriptive of the changes in the bias as function of the experienced load and, precisely, if the loading torque changes of 1 Nm the *Matching bias* is expected to change, with a confidence of 86%, of 10°. A possible explanation could be related to the idea that, as stretch receptors, muscle spindles increase their discharge rate proportionally to the amount of stretch imposed on the muscle (Allen and Westerblad, 2007). A higher rate is interpreted by the brain as a longer muscle thus resulting in a more flexed joint and, according to this theory, in case of higher effort (high resistive and viscous) targets were undershot since the brain interpreted muscles longer than they actually were (and therefore the joint more flexed than reality). Moreover, high resistive and viscous conditions were the ones in which participants experienced the highest forces, which probably prevented participants to reach the target position.

Influence of force in perception literature is still scarce and the existing studies (see Proske and Gandevia, 2009 for review) are still in doubt if claiming a pure central origin of force sense (Gandevia and McCloskey, 1976; Brooks et al., 2013) or a relevant contribution of stretch receptors afferents and peripheral coding (Luu et al., 2011). Our work sought to spread light in this direction, providing further information which may lead to solve such debated dichotomy. What we could conclude from our study is that the influence of force on proprioception is coded centrally, but peripheral receptors have a strong influence on the final perception as well. Furthermore, we believe that the results presented here could be the basis for further experiments, in which the magnitude of force could be tested and EMG signals might be analyzed. For instance, it would be worth to investigate the relationship between the amplitude of the error (in terms of under/over estimation) and the amplitude of the resistive force, in particular if there is a correlation among them and what is the nature of such connection.

It is also worth considering the effect of another type of mechanical perturbation of the wrist flexion/extension movements, namely the vibration of an antagonist muscle. Of course, this kind of perturbation involves different physiological mechanisms: strong illusions of movement in the case of vibration (Goodwin et al., 1972) and coupled force/position control in the present study. As a matter of fact, studying the JPM with different resisting force fields is not aimed at evaluating the effect of a disturbance but the degree of robustness of the JPS in typical loading conditions of everyday life. However, it is worth mentioning that in a previous study (Rickards and Cody, 1997) on the proprioceptive control of wrist movements, which included healthy subjects and Parkinson's disease patients, it was found that the main effect of muscle vibration was a vibration-induced undershoot during target reaching: a similar undershoot effect was also found in this study.

Furthermore, in the presented work, we proposed an analysis of wrist proprioceptive acuity with a robotic device to accurately quantify the influence of external force on wrist sensitivity by means of a reliable and repetitive protocol, easy to administer and which did limit the attention load for the subjects. Reliability of such experimental protocol was previously tested (Cappello et al., 2015; Marini et al., 2016a) and validated (Marini et al., 2016b, 2017) and the involved robotic device appeared to be a high suitable platform to assess wrist proprioceptive functions providing, clear, quantitative, and precise information about the joint sensitivity.

The method proposed in this work can be used to test the matching performance of patients presenting proprioceptive impairments. Other studies have recently shown that clinical features of dystonia differentially affect multi-sensory integration of visual and sense of effort signals and that force control deficits depend on the sensory information available (Carment et al., 2017). The result reported is in line with the optimal multi-sensory integration theory (Ronsse et al., 2009), claiming that subjects focus on the most reliable information among different sensory feedbacks. The method proposed in the present contribution can be used to test which, between positional information and sense of effort, is the most reliable feedback for patients affected by different pathological conditions.

To conclude, in the last few years haptic devices have been extensively used to implement novel methods to study proprioception, but most of the contributions considered either proximal upper limb joints [such as the elbow (King et al., 2013) and shoulder (Brindle et al., 2004; Erickson and Karduna, 2012)], or lower limb joints [such as the knee (Neufeld, 1981) and ankle (Domingo et al., 2014)] but, no studies have been conducted to provide reliable and quantitative data on how external forces influence wrist proprioceptive acuity. We therefore focused our attention on the distal arm, that is essential for human fine motor control, manipulation and the haptic perception of objects. The wrist is indeed involved in the execution of a wide variety of activities of daily living which can be compromised as a consequence of neurological damages, and a comprehensive knowledge about wrist proprioception especially in different dynamic conditions is a key information to consider for sensorimotor rehabilitation.

## Author contributions

FM conceived the idea and concept, designed and implemented the experiment, analyzed and interpreted the data, and drafted the manuscript. SC and CA helped in conceiving the idea and concept, designing and implementing the experiment, acquiring the data, interpreting the results and revising the manuscript. PM helped in drafting the manuscript and critically revising it. LM contributed in supervising the study. All authors read and approved the final manuscript.

### Conflict of interest statement

The authors declare that the research was conducted in the absence of any commercial or financial relationships that could be construed as a potential conflict of interest.
